# Audiovisual cues benefit recognition of accented speech in noise but not perceptual adaptation

**DOI:** 10.3389/fnhum.2015.00422

**Published:** 2015-08-03

**Authors:** Briony Banks, Emma Gowen, Kevin J. Munro, Patti Adank

**Affiliations:** ^1^School of Psychological Sciences, University of ManchesterManchester, UK; ^2^Faculty of Life Sciences, University of ManchesterManchester, UK; ^3^Speech, Hearing and Phonetic Sciences, University College LondonLondon, UK

**Keywords:** speech perception, perceptual adaptation, accented speech, audiovisual speech, multisensory perception

## Abstract

Perceptual adaptation allows humans to recognize different varieties of accented speech. We investigated whether perceptual adaptation to accented speech is facilitated if listeners can see a speaker’s facial and mouth movements. In Study 1, participants listened to sentences in a novel accent and underwent a period of training with audiovisual or audio-only speech cues, presented in quiet or in background noise. A control group also underwent training with visual-only (speech-reading) cues. We observed no significant difference in perceptual adaptation between any of the groups. To address a number of remaining questions, we carried out a second study using a different accent, speaker and experimental design, in which participants listened to sentences in a non-native (Japanese) accent with audiovisual or audio-only cues, without separate training. Participants’ eye gaze was recorded to verify that they looked at the speaker’s face during audiovisual trials. Recognition accuracy was significantly better for audiovisual than for audio-only stimuli; however, no statistical difference in perceptual adaptation was observed between the two modalities. Furthermore, Bayesian analysis suggested that the data supported the null hypothesis. Our results suggest that although the availability of visual speech cues may be immediately beneficial for recognition of unfamiliar accented speech in noise, it does not improve perceptual adaptation.

## Introduction

When we encounter a speaker with an unfamiliar accent, we are able to ‘tune in’ to the new phonetic patterns of speech to understand what they are saying. This type of perceptual adaptation is regularly encountered in daily life and allows us to recognize speech in a variety of native and non-native accents (Clarke and Garrett, 2004; Bradlow and Bent, 2008; Maye et al., 2008). It is a robust ability that is present in all stages of life (for a review, see Cristia et al., 2012) and occurs even with relatively unintelligible accents, albeit at a slower rate (Bradlow and Bent, 2008). The relative success and speed of perceptual adaptation depends on external factors such as the amount and variety of exposure to the accent (Bradlow and Bent, 2008). However, less is known about how the modality of speech can influence the adaptation process – for example, whether adaptation to accented speech is greater when audiovisual speech cues are available, compared to only auditory speech cues. Identifying ways to improve or facilitate this process may benefit communication in certain populations who have difficulty adapting to accented speech, such as older adults (Adank and Janse, 2010), individuals with aphasia (Bruce et al., 2012), or non-native speakers (Munro and Derwing, 1995); for example, audiovisual speech could be incorporated into language-learning tools or rehabilitation therapies for aphasia.

Perceptual adaptation to accented speech can be seen as a three-stage process: the listener first perceives the new, unfamiliar input; secondly, maps this onto stored lexical items, and thirdly, generalizes these new mappings to other lexical items. Indeed, research has successfully shown that this type of adaptation involves the modification of perceptual phonemic boundaries in relation to perceived lexical items (Norris et al., 2003; Kraljic and Samuel, 2005, 2006); for example, listeners who perceive an ambiguous sound midway between /d/ and /t/ spoken within the word ‘crocodile,’ are more likely to then categorize the same sound as /d/ when heard in isolation.

An improvement in perceptual adaptation to accented speech could potentially be achieved by influencing any one of the three stages involved, for example, the first stage may be facilitated through the availability of audiovisual (multisensory) cues. The integration of multisensory input across different sensory modalities can facilitate perception (Stein and Meredith, 1993); for example, auditory perception of speech is improved when integrated with visual input from a speaker’s facial movements. Indeed, being face-to-face with a speaker improves speech recognition in noisy environments (Sumby and Pollack, 1954; Erber, 1975; MacLeod and Summerfield, 1987; Grant et al., 1998; Ross et al., 2007), particularly when speech is non-native (Reisberg et al., 1987; Arnold and Hill, 2001; Hazan et al., 2006). Research has shown that audiovisual speech cues help listeners to identify fricative consonants (Jongman et al., 2003) and prosodic cues such as lexical prominence (Swerts and Krahmer, 2008). The benefits of audiovisual cues may also extend to accented speech, as several studies have shown that recognition of accented speech is better for audiovisual compared to audio-only input (Arnold and Hill, 2001; Janse and Adank, 2012; Yi et al., 2013; Kawase et al., 2014). The integration of auditory and visual cues may benefit recognition of accented speech by helping listeners to resolve the perceptual ambiguities of an unfamiliar accent; for example, if a speaker’s pronunciation of a particular phoneme or word is unclear, observing their mouth movements may help to identify the correct item. Indeed, exposure to ambiguous audiovisual cues using McGurk stimuli has been shown to influence subsequent phoneme categorization (Bertelson et al., 2003; Vroomen et al., 2004). A listener who is face-to-face with an accented speaker may therefore be able to exploit the perceptual benefit from additional visual input, and adapt more successfully to the accented speech – that is, their recognition of the speech may improve more greatly over time.

Although a large part of everyday communication is carried out face-to-face, most experimental work on accent perception is carried out in the auditory modality, and the use of visual speech information has gained relatively little attention in relation to perceptual adaptation to accented speech. Furthermore, much of the work regarding the potential benefits of audiovisual speech to perceptual adaptation has been carried out using noise-vocoded speech rather than accented. While both speech types are less intelligible than familiar speech, and listeners adapt to them both, variation in noise-vocoded speech stems from degrading the acoustical composition of the entire speech signal, whereas accented speech varies in terms of its phonemic patterns, is acoustically intact and only affects certain speech sounds. Although audiovisual cues have been shown to benefit perceptual adaptation to noise-vocoded speech (Kawase et al., 2009; Pilling and Thomas, 2011; Wayne and Johnsrude, 2012; Bernstein et al., 2013), the observed effects are relatively small and, furthermore, we do not know if such results generalize to accented speech.

Two previous studies have investigated the role of audiovisual cues in perceptual adaptation to accented speech. In a phoneme-recognition study, Hazan et al. (2005) demonstrated that long-term perception of individual non-native phonemes improved when listeners were exposed to audiovisual input, compared to audio-only input; however, this finding was not tested with longer items such as sentences, and it is thus unclear if the results can be generalized to non-native speech in general. Indeed, when Janse and Adank (2012) compared perceptual adaptation to unfamiliar, accented sentences with or without visual cues, they observed no difference in the amount of adaptation, although a small, non-significant trend of greater adaptation during the early stages was present for audiovisual speech. However, two confounding factors may have influenced their findings. The experiment was carried out on older adults, a population that can have particular difficulty with processing visual speech (Sommers et al., 2005); this factor, combined with a relatively difficult semantic verification task, may have rendered the task cognitively demanding for the older participants and negatively affected their performance. Two possible conclusions can therefore be drawn from the two studies described here: first, audiovisual speech cues are not beneficial to perceptual adaptation to longer items of accented speech, although they may improve learning of particular phonemes in isolation (as shown by Hazan et al., 2005); or, audiovisual speech cues do benefit perceptual adaptation to accented speech, but the confounding factors outlined above prevented this effect from being observed. Therefore, evidence from young, healthy adults, using whole sentences and a simple speech recognition task, may help to establish the possible benefits of audiovisual speech cues for perceptual adaptation to accented speech.

We investigated whether audiovisual speech cues do indeed facilitate perceptual adaptation to accented speech. We did this across two studies, each using a different accent and speaker and a different experimental design, but with the same sentences and task. In particular, Study 2 addresses a number of questions arising from Study 1 (see Discussion, Study 1 for details). Study 1 employed a training design similar to those used in studies of noise-vocoded speech (Kawase et al., 2009; Pilling and Thomas, 2011; Wayne and Johnsrude, 2012), and a novel accent to control for familiarity effects (Maye et al., 2008; Adank and Janse, 2010; Janse and Adank, 2012). Participants underwent training in the novel accent with audiovisual or audio-only stimuli, with or without background noise. A visual-only (speech-reading) training condition provided a control group; that is, we did not expect visual training to affect adaptation to the accented speech. For the pre- and post-training sessions, we presented our accented stimuli in background noise to avoid ceiling effects associated with rapid perceptual adaptation (Janse and Adank, 2012; Yi et al., 2013). We also included two training conditions with background noise for two reasons: firstly, the learning context can influence the outcome of learning (Godden and Baddeley, 1975; Polyn et al., 2009), and consistency between the training and subsequent testing sessions may therefore affect adaptation. As the stimuli in our pre- and post-training sessions were always presented in the context of background noise, we predicted that training with background noise would facilitate recognition of the accented speech in noise following the training. Secondly, we predicted that altering the clarity of the auditory signal (by adding background noise) would increase the use of visual cues during the training (cf. Sumby and Pollack, 1954), and that this would, in turn, increase subsequent adaptation.

If audiovisual cues are beneficial to perceptual adaptation to accented speech, we expected to observe the following: (1) greater adaptation after audiovisual training compared to audio-only or visual-only training; (2) greater adaptation after audiovisual training with background noise compared to audiovisual training in quiet; (3) a greater ‘audiovisual benefit’ (the difference in adaptation between audiovisual and audio-only training) for the groups trained with background noise, compared to the groups trained without background noise; (4) greater adaptation following all types of training in comparison to visual training (that is, we expected the visual training to have no effect on subsequent recognition of the accented speech). Based on previous evidence that audiovisual cues can benefit recognition of accented speech compared to audio-only cues (Arnold and Hill, 2001; Janse and Adank, 2012; Yi et al., 2013; Kawase et al., 2014), we also expected to observe the following during the training session: (1) better recognition of the accented training stimuli for both audiovisual groups compared to the audio-only groups; (2) poorer recognition of the training stimuli presented in background noise compared to quiet; and (3) poorer recognition of the visual training stimuli compared to all other groups.

In Study 2, participants listened to a non-native (Japanese) accent in the audiovisual or auditory modality to test whether a greater amount of *continuous* exposure to audiovisual stimuli (without separate training) would reveal a difference in adaptation between the two modalities. This design enabled us to examine the overall amount of adaptation, as well as adaptation at different time points in the experiment (for example, the presence of audiovisual speech cues may afford benefits to recognition of accented speech in earlier compared with later trials; Janse and Adank, 2012). In addition, participants’ eye movements were recorded to verify that they were predominantly looking at the speaker’s face. As in Study 1, if audiovisual cues *are* beneficial to perceptual adaptation to accented speech, we predicted that participants exposed to audiovisual accented speech would adapt to a greater extent than participants exposed to audio-only accented speech. Conversely, if audiovisual cues *are not* beneficial to perceptual adaptation to accented speech, we expected to observe no difference in perceptual adaptation for the audiovisual and auditory modalities in either study.

## Study 1

### Methods

#### Participants

One hundred and five students (26 male, *Median* = 20 years, age range 18–30 years) recruited from the University of Manchester, participated in the study. All participants were native British English speakers with no history of neurological, speech or language problems (self-declared), and gave their written informed consent. Participants were included if their corrected binocular vision was 6/6 or better using a reduced Snellens chart, and their stereoacuity was at least 60 s of arc using a TNO test. Participants’ hearing was measured using pure-tone audiometry for the main audiometric frequencies in speech (0.5, 1, 2, and 4 kHz) in both ears. Any participant with a hearing threshold level greater than 20 dB for more than one frequency in either ear was excluded and did not participate in the study. We excluded one male participant based on the criteria for hearing, and four (one male, three female) based on the criteria for vision. We provided compensation of course credit or £7.50 for participation. The study was approved by The University of Manchester ethics committee.

#### Materials

We used 150 Institute of Electrical and Electronics Engineers (IEEE) Harvard sentences (IEEE, 1969) for our stimuli, and a 30-years-old male volunteer provided all recordings for the experiment. We transcribed and recorded 135 sentences in the novel accent, and randomly divided them into three lists, A, B or C. We recorded the remaining 15 sentences in the speaker’s own British English accent to provide stimuli for a ‘familiar accent’ baseline test. We used a novel accent to avoid confounds from participant familiarity (that is, we could guarantee that none of our participants had ever encountered it before; see Adank et al., 2009), and to compare responses to the novel, unfamiliar accent with a familiar accent (our baseline measurement) from the same speaker (Adank and Janse, 2010). The novel accent (see Banks et al., 2015 for further details) was created by systematically modifying the vowel sounds of a Standard British English accent (**Table 1**). The accent was created using allophones from existing regional English accents (for example, Scottish or Irish) through an iterative process.

**Table 1:** Phonetic description of the novel accent.

IPA	Example
ɪ → ɛ	sit → set
ɛ → ɪ	bet → bit
æ → ɛ	hat → het
∧ → ʊ	cud → could
зː → ɛə	girl → gairl
aː → ɔː	dark → dork
ɒ → ɔː	hot → hawt
ɔː	door
uː	food
ʊ	good
ə	mother
iː	tree
ɛə → зː	hair → her
əʊ → aʊ	vote → vowt
aʊ → uː	how → hoo
ɛɪ → aɪ	way → wye
aɪ → ɔɪ	my → moy
ɪə	hear
ɔɪ	joy

##### Training stimuli

Stimuli for the training sessions comprised six movies (three with and three without background noise), each comprising 45 video clips from one of the three novel-accented stimuli lists (A, B, and C). During recordings, the speaker looked directly at the camera with a neutral expression, and was asked to speak as naturally as possible. The recordings were made in a sound-treated laboratory with no natural light, using a High Definition Canon HV30 camera and Shure SM58 microphone. The camera was positioned ~1 m from the speaker to frame the head and shoulders, with a blue background behind the speaker. Video recordings were imported into iMovie 11, running on an Apple MacBook Pro, as large (960 × 540) digital video (.dv) files. Each recorded sentence was edited to create a 6-s video clip which were then compiled in a randomized order to create the training videos. Between each clip (sentence) there was a 7-s interval, during which the screen was black with a white question mark for 4 s (to indicate to participants they should respond) and a white fixation cross for 3 s (to indicate the next clip was imminent). Edited audio files (see Testing Stimuli, below) were re-attached to each video clip so that the normalized stereo tracks would be heard congruently with the video. For training conditions that included background noise, we added speech-shaped noise at a signal-to-noise ratio (SNR) of 0 dB to the audio files, using a custom script in Matlab software (R2010a, Mathworks, Inc.), before re-attaching them. Each movie was exported as a 960 × 540 MPEG-4 movie file with a bit-rate of 3269, in widescreen (16:9) ratio at 25 frames per second.

##### Testing stimuli

The audio track for each video clip (sentence) was extracted as an audio (.wav) file to be used for the auditory testing sessions. The experimenter checked all recordings and any that were not deemed suitable (for example due to mispronunciation or unnaturalness) were re-recorded in a second recording session. Audio files were normalized by equating the root mean square amplitude, resampled at 22 kHz in stereo, and cropped at the nearest zero crossings at voice onset and offset, using Praat software (Boersma and Weenink, 2012). The same procedure was used for the native-English recordings to produce stimuli for the familiar-accent baseline test.

We counterbalanced the presentation order of the novel-accented stimuli for the pre-training, training and post-training sessions across training groups; this was based on the sentence lists and followed the order ABC, CAB, and BCA. Each sentence was presented once per participant to avoid item-specific training effects. During the pre-training and post-training sessions, sentences were presented in a pseudo-random order per testing block and per participant, and the sentences used for the baseline and training sessions were presented in a fixed order.

#### Procedure

**FIGURE 1:**
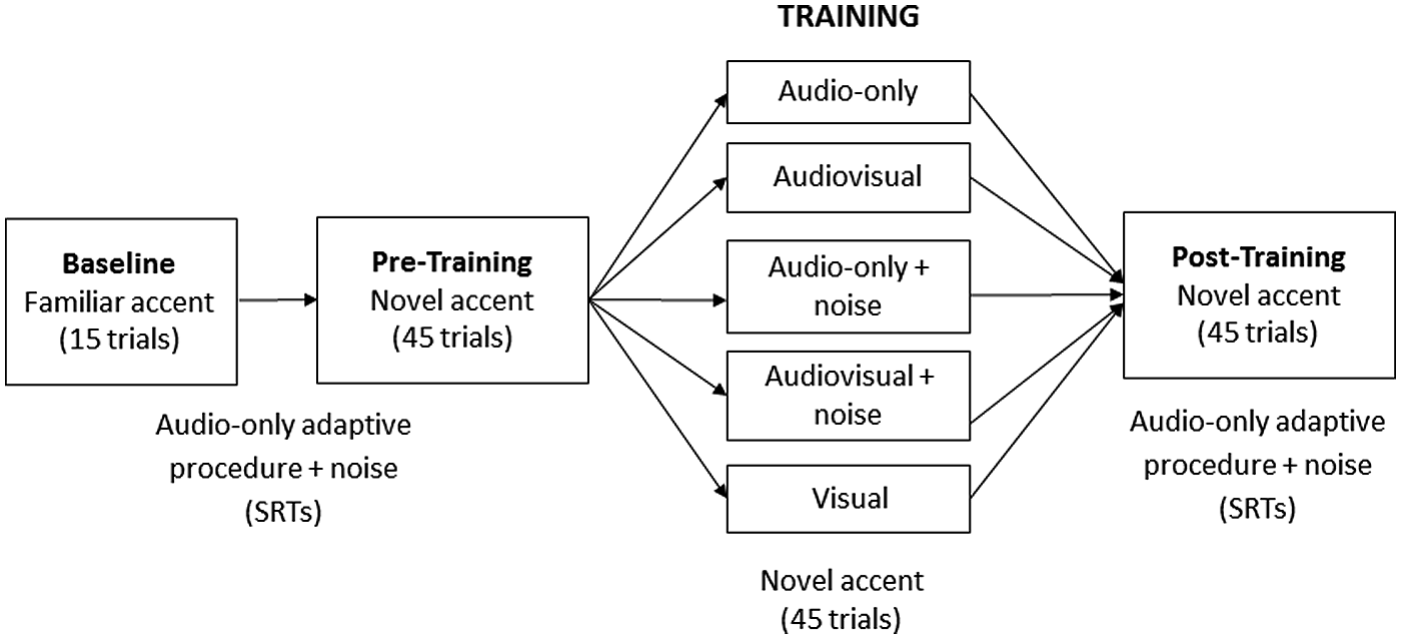
**Design for Study 1.** The baseline session comprised 15 familiar-accented sentences; the pre-training, training and post-training session comprised 45 novel-accented sentences each.

**Figure 1** shows the experimental design in full. Participants first listened to the 15 familiar-accented (baseline) sentences to habituate them to the task and to the background noise. This was followed by the pre-training session, after which participants underwent training in one of five randomly assigned conditions (*N* = 20 per group): audiovisual, audio-only, visual (speech-reading), audiovisual + noise, audio-only + noise. Each participant was exposed to training stimuli from one of the three lists (A, B, or C) presented on a laptop computer. However, for the two audio-only groups the screen was not visible, and for the visual group, participants were asked to remove their headphones and to speech-read each sentence. Each session (pre-training, training, and post-training) comprised 45 sentences.

##### Speech reception thresholds

For the baseline, pre-training and post-training sessions (but not for the training), we measured participants’ recognition accuracy as speech reception thresholds (SRTs; Adank and Janse, 2010; Banks et al., 2015) in speech-shaped background noise, a sensitive measure which eliminates the need to equate starting accuracy between participants as it keeps recognition accuracy constant throughout. An adaptive staircase procedure (Plomp and Mimpen, 1979) varied the SNR per trial depending on the participants’ response; that is, the SNR increased following an incorrect response, decreased following a correct response, or remained constant if a response was 50% correct. Thus, the SNR decreased as participants’ performance improved (Baker and Rosen, 2001). The SNR varied in pre-determined steps of 8 dB for the first two changes and 2 dB thereafter, and maintained recognition accuracy (number of correctly repeated keywords) at 50%. The procedure was carried out using Matlab (R2010a). The mean SNR for all reversals indicated the SRT measurement for each participant, with an average of 21 reversals (SD = 5.4) per 45 trials.

##### Speech recognition task

Throughout the experiment, we instructed participants to repeat out loud as much of each sentence as they could in their normal voice and without imitating the accent. The experimenter scored participants’ responses immediately after each trial, according to how many keywords (content or function words) they correctly repeated out of a maximum of four (for example, “a pot of tea helps to pass the evening”). Responses were scored as correct despite incorrect suffixes (such as -s, -ed, -ing) or verb endings; however, if only part of a word (including compound words) was repeated this was scored as incorrect (Dupoux and Green, 1997; Golomb et al., 2007; Banks et al., 2015). If a participant imitated the novel accent rather than responding in their own accent this was also scored as incorrect, as we could not make a clear judgment as to whether they had recognized the correct word.

All tests and training were carried out in a quiet laboratory in one session lasting ~50 min. Auditory stimuli for the baseline and testing sessions were presented using Matlab software (R2010a, Mathworks, Inc.), and training stimuli were presented using iTunes 10.5.1 on an Apple MacBook Pro. Participants wore sound-attenuating headphones (Sennheiser HD 25-SP II) for the duration of the experiment, except during the visual (speech-reading) training. The experimenter adjusted the volume to a comfortable level for the first participant and then kept it at the same level for all participants thereafter.

#### Data Analysis

Perceptual adaptation was defined as the difference in SRTs before and after the training. We carried out a mixed-design ANOVA with a within-participant factor of testing session (two levels: pre- and post-training), and a between-group factor of training type (five levels: audio-only, audiovisual, visual-only, audio-only + noise, audiovisual + noise), was conducted on these difference scores. To investigate recognition of the novel accent in the different training modalities, we also analyzed accuracy scores (% correct keywords) from *within* the training session by conducting a one-way ANOVA (five levels: audio-only, audiovisual, visual-only, audio-only + noise, audiovisual + noise). To verify that baseline and pre-training measurements were equal across all groups, we carried out a one-way ANOVA for each data set with the between-group factor of training group (five levels). All *post hoc t*-tests carried out were two-tailed and we applied a Bonferroni correction for multiple comparisons. We identified two outliers in the data (one for the novel-accented SRTs and one for the baseline SRTs) with standardized residuals >3.29^1^, and these scores were modified to the value of the group mean SRT plus two standard deviations. Unless otherwise stated, our data met all other assumptions for the parametric tests that we used.

### Results

**FIGURE 2:**
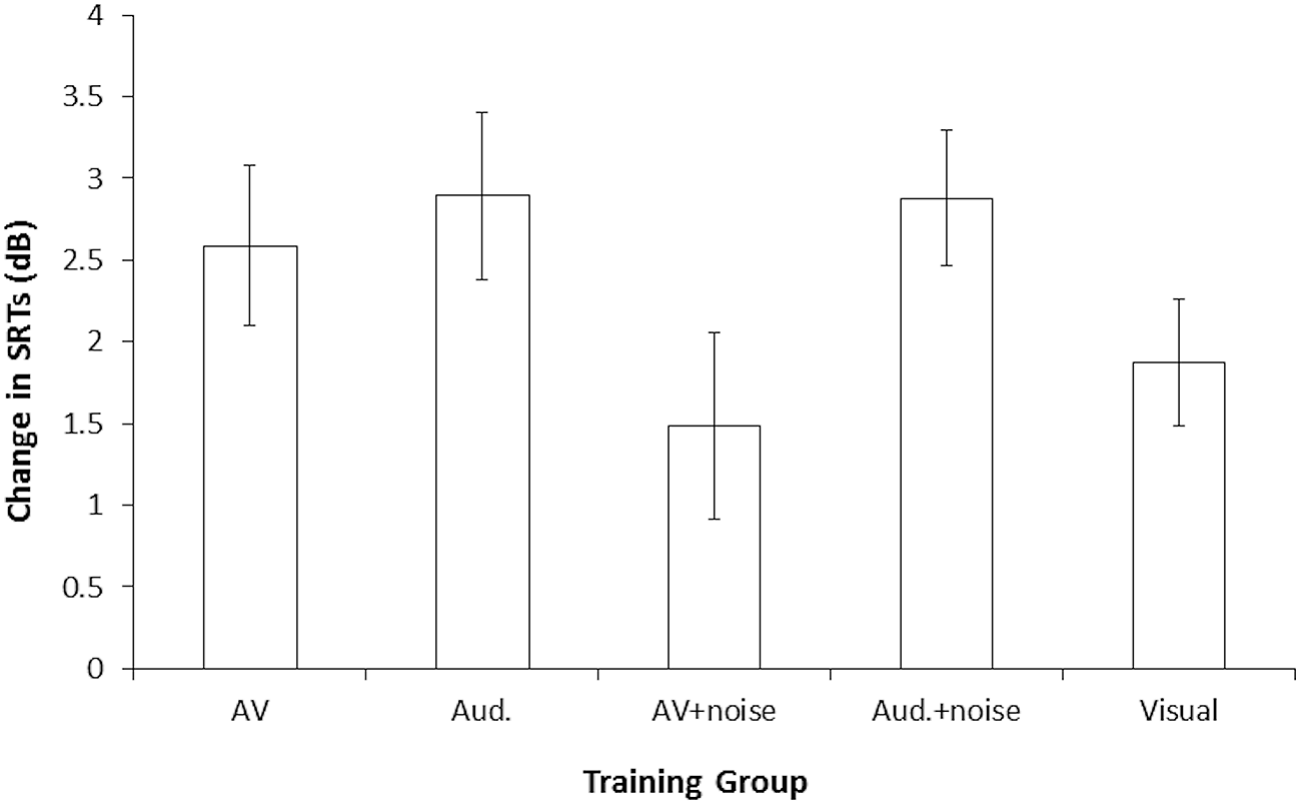
**Amount of perceptual adaptation to accented speech in Study 1: mean improvement in SRTs following training, per group (a higher change in SRTs indicates greater improvement).** Error bars represent ± 1 SE. AV, audiovisual; Aud., audio-only.

**FIGURE 3:**
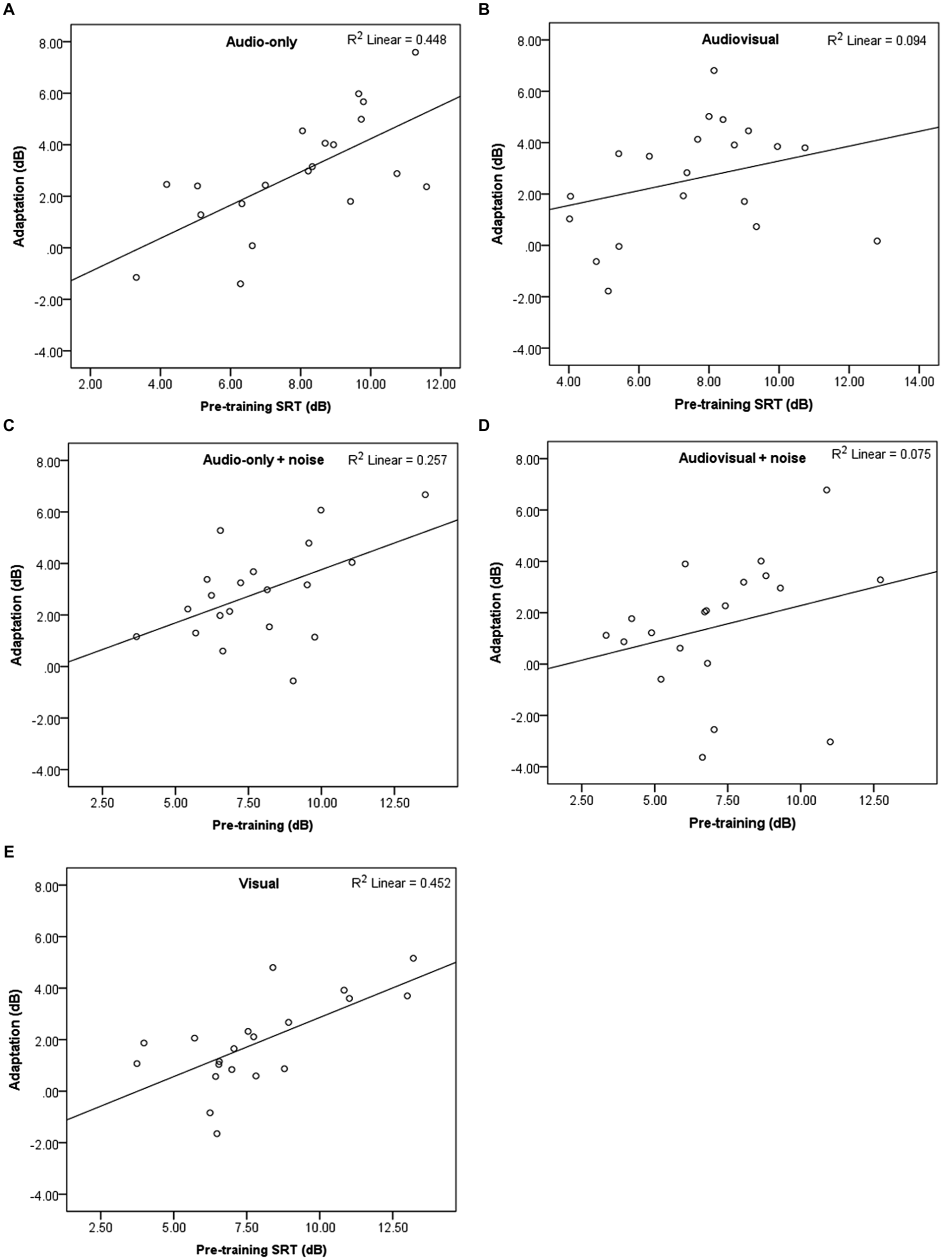
**(A–E)** Scatterplots showing pre-training SRTs and amount of adaptation with linear fit, per group (Study 1).

**Table 2** shows the mean SRTs for the familiar-accented (baseline) speech, and mean pre- and post-training SRTs for the novel accent, per training group. As SRTs represent the SNR (dB) at which 50% recognition accuracy is achieved, higher levels reflect poorer performance. SRTs in all groups decreased following the training by ~2 dB, indicating that participants’ recognition of the accented speech improved over time and that perceptual adaptation took place. **Figure 2** shows the mean decrease in SRTs (amount of perceptual adaptation) following the training for each group. **Figures 3A–E** show a negative relationship between the amount of adaptation and pre-training SRTs; that is, participants who initially performed relatively worse adapted the most.

**Table 2:** Mean SRTs in dB per training group (Study 1).

	Familiar accent		Novel accent					
	Baseline SRT		Pre-training SRT		Post-training SRT	
Training group	*M*	SD	*M*	SD	*M*	SD
Audiovisual	0.4	1.68	7.6	2.33	5.0	2.66
Audio-only	0.2	1.42	7.9	2.36	5.0	1.88
Visual	0.6	1.75	7.8	2.56	6.0	1.89
Audiovisual + noise	0.9	2.19	7.4	3.13	5.9	3.46
Audio-only + noise	0.7	1.45	7.9	2.28	5.0	2.08
All groups	0.6	1.70	7.7	2.50	5.4	2.47

*Training group *N* = 20.*

No significant differences were observed between groups for baseline SRTs (recognition of familiar-accented speech), or for pre-training SRTs (recognition of the novel-accented speech), confirming that the groups were equally matched for comparison. As expected, baseline SRTs across all five groups (*M* = 0.5 dB, SD = 1.68) were significantly lower than mean pre-training SRTs, across all groups (*M* = 7.7 dB, SD = 2.50), *t*(99) = 29.19, *p* < 0.001, confirming that the novel accent negatively affected participants’ recognition in comparison to the familiar accent. We observed a main effect of testing session, *F*(1,95) = 119.48, *p* < 0.001, $\eta_{p}^{2}$ = 0.56. Paired-sample *t*-tests (Bonferroni correction, *p* < 0.01) confirmed that decreases in SRTs following the training were statistically significant in every group (see **Table 2**); thus, participants’ recognition of the accented speech significantly improved between the two sessions. Neither the main effect of training group, nor the testing session × training type interaction, were significant (*p*s > 0.05).

A null finding may be interpreted in two ways: (1) that no effect is present in the population and the null hypothesis is true, or (2) that the data are inconclusive; however, significance testing cannot confirm these interpretations. Calculating Bayes factor (*B*) can, however, test whether the null hypothesis is likely, regardless of observed *p*-values. We calculated Bayes factor for differences in the amount of adaptation between all five groups (see **Figure 2** and **Table 3**). These analyses indicated that the null hypothesis (that there was no difference in adaptation between the groups) was supported for the following comparisons: audiovisual vs. audio, audiovisual vs. visual, audiovisual + noise vs. visual, audiovisual vs. audio + noise, and audio vs. audio + noise (*B* < 0.33; significant differences between these groups were predicted if our experimental hypotheses were true). All other comparisons indicated that data from this sample were inconclusive (0.33 < *B* < 3.0).

**Table 3:** Bayes factor (*B*) for comparisons of adaptation between groups in Study 1.

Group	AV	Audio	Visual	AV + noise
AV	–			
Audio	0.21*	–		
Visual	0.43	0.90	–	
AV + noise	0.85	1.68	0.24*	–
Audio + noise	0.20*	0.14*	1.10	1.97

N = 20 per group. Calculations based on a uniform distribution with lower and upper limits of 0–6 dB change. *B* < 0.33 indicates results in favor of the null hypothesis; *B* > 3 indicates results in favor of the experimental hypothesis; intermediate values indicate that data is inconclusive. *B < 0.33.

#### Analysis of the Training Data

**FIGURE 4:**
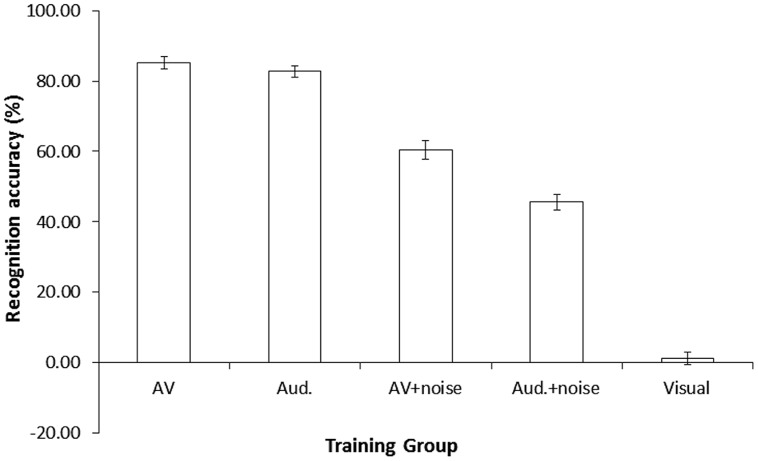
**Mean % recognition accuracy of accented speech per group, during the training session in Study 1.** Error bars represent ± 1 SE. AV, audiovisual; Aud., audio-only.

To further investigate how the presence of audiovisual cues affected participants’ recognition of the novel accent, we analyzed recognition accuracy in the five groups *during* the training (**Figure 4**). Analysis of these data revealed a significant effect of training condition, *F*(4,95) = 331.47, *p* < 0.001, $\eta_{p}^{2}$ = 0.93. Pairwise comparisons (Bonferroni correction, *p* < 0.005) confirmed that recognition accuracy was significantly lower in the visual group (*M* = 1.4%, SD = 0.82) than in all other groups, *p* < 0.001. Recognition accuracy was also significantly higher in the audiovisual (*M* = 85.2%, SD = 7.67) and audio-only (*M* = 82.7%, SD = 7.17) groups compared to the audiovisual + noise (*M* = 60.4%, SD = 11.90) and audio-only + noise (*M* = 45.6%, SD = 10.01) groups, *p*s < 0.001. Recognition accuracy was significantly higher in the audiovisual + noise compared to the audio-only + noise group, *p* < 0.001. However, the marginal difference between the audiovisual and audio-only groups was not statistically significant, *p* = 0.289, and a Bayes factor calculation suggested that the data were inconclusive, *B* = 0.30 (uniform distribution, 0–30% limit).

### Discussion

In Study 1, we investigated whether training with audiovisual or audio-only speech, with or without the presence of background noise, affected perceptual adaptation to a novel accent. As in previous studies of perceptual adaptation to accented speech (Clarke and Garrett, 2004; Bradlow and Bent, 2008; Maye et al., 2008; Adank and Janse, 2010; Gordon-Salant et al., 2010; Janse and Adank, 2012), we observed significant improvements in recognition of the novel accent over time, represented by a decrease in SRTs following the training.

Contrary to our predictions, there was no significant difference in the amount of adaptation between any of the groups; that is, the type of training had no effect on adaptation. Bayes factor suggested that non-significant differences in adaptation for four of the group comparisons (most importantly, audiovisual vs. audio-only) supported the null hypothesis. This would suggest that audiovisual cues do not benefit adaptation to accented speech better than audio-only or visual-only stimuli. However, for most of the group comparisons (particularly audio-only vs. visual), Bayes factor indicated that the data were inconclusive. We had included visual training as a control group, and predicted that training with audio-only stimuli would lead to greater adaptation in comparison – this would indicate that the training had been effective. However, the difference between these groups was inconclusive, and we therefore cannot ascertain whether the training was fully effective, or whether the lack of differences between groups was due to methodological reasons.

Analysis of data from the training session confirmed our predictions that recognition accuracy for the visual group would be considerably and significantly lower than all other groups, and that audiovisual cues would provide a benefit to recognition of the accented speech, as recognition accuracy was significantly higher in the audiovisual + noise group than in the audio-only + noise group. However, the same ‘audiovisual benefit’ was not present for participants carrying out training in quiet, although this null-effect was inconclusive – perhaps because accuracy was almost at ceiling level for these groups (Ross et al., 2007). Nevertheless, any effects observed during the training did not transfer to subsequent auditory testing, again suggesting that the training was not fully effective.

There are several possible explanations for this. Firstly, the timing of the training, and the length of the pre-training session, meant that participants had already begun adapting to the novel accent before the training. The training may therefore not have been fully beneficial at this stage. With longer exposure to the audiovisual stimuli at an earlier time point, we may have observed an effect of greater adaptation for this group. Secondly, inconsistency between the training and subsequent testing sessions may have affected any benefits from the training, as consistency between training and subsequent testing can be beneficial to performance (Godden and Baddeley, 1975; Polyn et al., 2009). In fact, the switch to a separate training session may have been disruptive to adaptation. Thirdly, audiovisual cues from the particular speaker, or for the particular accent we used, may not have been sufficiently beneficial to improve perceptual adaptation. The relative benefit from audiovisual cues varies between different speakers (Kricos and Lesner, 1982, 1985), and this may also be the case for different accents. Indeed, Kawase et al. (2014) demonstrated that audiovisual cues vary in how much they can benefit recognition of non-native phonemes, in some cases even inhibiting recognition. Furthermore, Hazan et al. (2005) observed greater adaptation after audiovisual compared to audio-only training for *non-native* phonemes, whereas our novel accent was based on native (regional) English accents. We may therefore have observed a greater benefit to perceptual adaptation with audiovisual cues from a different speaker, and with a non-native accent.

To answer these remaining questions, we carried out a second study using a different experimental design, accent and speaker. In Study 2, we exposed participants to 90 sentences of unfamiliar accented speech in either the audiovisual or auditory modality without separate training, thus addressing concerns that the timing and length of the training, or inconsistency between training and testing sessions, affected the benefits gained from audiovisual cues in Study 1. Furthermore, this design allowed us to analyze the effects of audiovisual cues on adaptation at different stages of the experiment, for example during early compared with later trials, which may reveal more subtle effects (Janse and Adank, 2012). Secondly, we used a natural, non-native (Japanese) accent produced by a different speaker for our stimuli. Additionally, we recorded participants’ eye movements using an eye-tracker to verify that they were continually looking at the speaker’s face during testing. We increased the number of participants in each group to address any potential concerns that sample size prevented the effects in Study 1 from reaching statistical significance. By addressing these remaining questions, we hoped to clarify whether audiovisual speech cues can indeed benefit perceptual adaptation to unfamiliar accented speech.

## Study 2

### Methods

#### Participants

Sixty five young adults (five male, *Median* = 20.55 years, age range 18–30 years) recruited from the University of Manchester participated in the study, following the same procedure and exclusion criteria as Study 1. Two participants were excluded (one male, one female) due to data loss during the eye-tracking procedure (see Data Analysis for full details), and one female participant was excluded due to technical issues during the experiment.

#### Materials

Stimulus material consisted of 120 of the IEEE Harvard sentences (IEEE, 1969) that had been used in Study 1. A 30-year-old male native Japanese speaker recited 90 of them in a soundproofed laboratory, and these were recorded and edited using the same equipment and procedure as for Study 1. Speech-shaped background noise was added to the audio files using a custom Matlab script to create stimuli at SNRs of +4 to -4 dB. Background noise was included throughout to avoid ceiling effects associated with rapid perceptual adaptation to an unfamiliar accent (for example, Clarke and Garrett, 2004). For the audiovisual condition, the audio files were combined with the corresponding video clips using Experiment Builder software (SR Research, Mississauga, ON, Canada) to create congruous audiovisual stimuli. For the audio-only condition, a different static image of the speaker, taken from the video recordings, was displayed on screen simultaneously with each audio recording; this was to ensure that participants were processing auditory and visual information in both conditions. All stimuli were presented in a randomized order for each participant.

The native-accent baseline stimuli comprised the same 15 standard British English sentences from Study 1, plus an additional 15 recorded by the same speaker. We used 30 sentences to ensure that participants habituated to the background noise and task, as the SRT from this test would be used to set the SNR for presentation of the non-native accented stimuli. The baseline sentences were presented in a fixed order for all participants.

#### Procedure

All tests were carried out in a soundproofed booth in one session lasting ~40 min. The familiar-accented baseline stimuli were presented and scored using Matlab software (R2010a, Mathworks, Inc.), through Sennheiser HD 25-SP II headphones, in the same adaptive staircase procedure used in Study 1 (see Speech Reception Thresholds for details). An Eyelink 1000 eye-tracker with Experiment Builder software (SR Research, Mississauga, ON, Canada) was used to present the accented stimuli and to record participants’ eye movements. Participants wore the same headphones for the duration of the experiment, and sat with their chin on a chin rest facing the computer monitor. The experimenter adjusted the chin rest so that each participant’s eyes were level with the top half of the display screen, which was positioned 30 cm from the chin rest. Eye movements were recorded by tracking the pupil and corneal reflection of the right eye at a sample rate of 1000 Hz. Calibration was carried out using a standard 9-point configuration before the start of the experiment, and 5 min after the start time. A drift-check was carried out immediately before each trial and calibration was performed again if required.

Participants were randomly allocated to either the audiovisual (*N* = 32) or audio-only (*N* = 30) condition. The experimenter set the volume for all stimuli at a comfortable level for the first participant, and kept it at the same level for all participants thereafter. Participants first listened to the 30 native-accented baseline sentences. The SRT acquired for this test was then used to set the SNR at which the accented stimuli were presented in the background noise, for each individual participant. The SRT was rounded to the nearest whole number (for example, if a participant’s SRT for the familiar-accented speech was -1.3 dB, the SNR for the accented stimuli was set at -1 dB). This was intended to equate baseline recognition for the audiovisual group at ~50% accuracy; however, we expected recognition to be lower for the audio-only group. This would allow us to verify the amount of ‘benefit’ provided by the audiovisual speech. In both conditions, participants were requested to watch the screen and to repeat each sentence following the same task and scoring procedure as in Study 1. Oral responses were recorded using a Panasonic lapel microphone attached to the chin rest, and responses were scored retrospectively by the experimenter. All 90 accented sentences were presented consecutively, and participants pressed the space bar to trigger each trial at their own pace.

#### Data Analysis

We measured recognition accuracy by calculating % correctly repeated keywords per sentence. To compare recognition accuracy between groups, and to analyze changes over time, we fitted a linear function to each participant’s recognition data (Erb et al., 2012; Banks et al., 2015) using the equation *y = mx+b*, where *y* is the mean SRT, *x* is time (trial), *m* is the slope, and *b* is the intercept. The intercept of each participant’s linear fit was used as the measurement of recognition accuracy, and the slope was used as the measurement of adaptation. We carried out *t*-tests and Bayes factor calculations to analyze effects of modality on recognition accuracy and perceptual adaptation. To confirm that participants in the audiovisual group were predominantly looking at the speaker’s face, we created a semi-circular region of interest around this area, and calculated percent fixation time in this region for the duration of the stimulus presentation. We analyzed eye-tracking samples to check for data loss (for example due to blinks or head movements); trials with >20% data loss were excluded, and two participants who had >5 trials excluded were not included in our analyses (number of excluded trials: *M* = 1.24, SD = 3.06). For consistency, eye movement data were collected for both groups; however, as the data from the audio-only group is not relevant to this paper, these data will not be discussed further. All other analyses were conducted in the same way as in Study 1.

### Results

**FIGURE 5:**
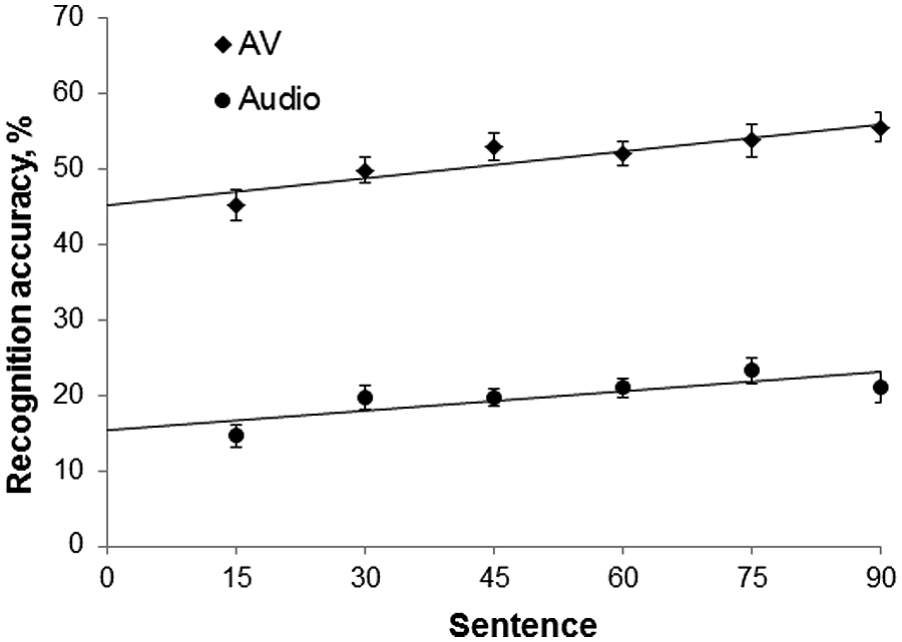
**Mean % recognition accuracy of accented speech in Study 2, per 15 sentences, per group, with linear fit.** Error bars represent ± 1 SE. AV, audiovisual; Audio, audio-only.

**FIGURE 6:**
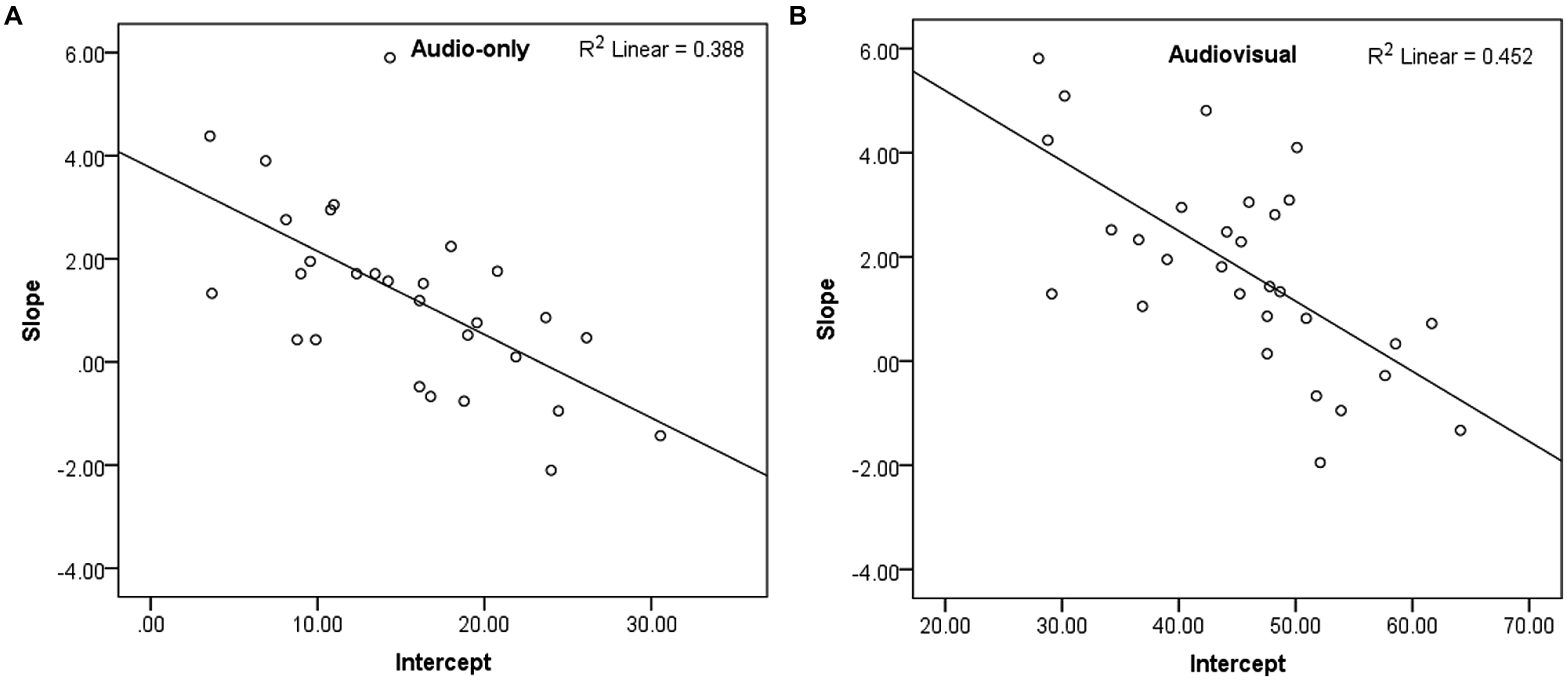
**(A,B)** Scatterplots showing slope (adaptation) and intercept (baseline recognition accuracy) for recognition of the accented speech in Study 2, per group and with linear fit.

**Figure 5** shows mean recognition accuracy of the accented speech in the audiovisual and audio-only modalities, with linear fits. Recognition accuracy increased over time by a maximum of 10.8% (SD = 10.94) in the audiovisual group, and a maximum of 8.7% (SD = 13.61) in the audio-only group, suggesting that both groups adapted to the non-native accented speech. Recognition accuracy was consistently greater in the audiovisual group than the audio-only group, with a difference of ~30% between the groups throughout the experiment. An independent-samples *t*-test confirmed that there was no significant difference in native-accented SRTs between the two groups, and that they were equally matched in their baseline ability to process non-native speech in background noise. **Figures 6A,B** show a negative relationship between the slope and intercept in each group indicating that, as in Study 1, participants with lower starting accuracy adapted the most.

There was a significant difference in the intercept for the audiovisual group (*M* = 45.32, SD = 9.52) and the audio-only group (*M* = 14.44, SD = 6.82); *t*(57) = 13.82, *p* < 0.001, *d’* = 3.58, confirming that recognition accuracy was significantly greater for the audiovisual group. However, there was no significant difference in slope between the audiovisual group (*M* = 1.78, SD = 1.91) and the audio-only group (*M* = 1.27, SD = 1.77), *t*(57) = 1.07, *p* = 0.291, *d’* = 0.28. A Bayes factor calculation confirmed that the null hypothesis (that there was no difference in adaptation between the two groups) was likely, *B* = 0.09 (based on a uniform distribution and upper and lower limits of 0–20% improvement). Finally, analysis of the eye-tracking data confirmed that participants primarily looked at the speaker’s face during presentation of the audiovisual stimuli (% gaze time on the speaker’s face: *M* = 100%, SD = 0.01%).

### Discussion

Study 2 investigated whether perceptual adaptation to non-native accented speech differed when participants were exposed to audiovisual or audio-only stimuli. In comparison to Study 1, we exposed participants to the accented stimuli in either the audiovisual or audio-only modality without separate training. Participants were now exposed to twice as many audiovisual sentences as the training groups in Study 1, and could potentially benefit from the audiovisual cues at all stages of the experiment. Participants also performed the task in consistent conditions throughout the experiment without interruption, rather than in different modalities for testing and training. We used a Japanese accent and a different speaker for our stimuli to test whether audiovisual cues were more beneficial for recognizing a non-native accent (in comparison to the novel accent used in Study 1). Lastly, we recorded participants’ eye gaze to confirm that they looked predominantly at the speaker’s face.

As in Study 1, recognition accuracy of the accented speech significantly improved over time. We observed a maximum increase of ~10%, which is similar to previous studies of perceptual adaptation to accented speech (Bradlow and Bent, 2008; Gordon-Salant et al., 2010; Janse and Adank, 2012). As predicted, participants exposed to audiovisual stimuli had better overall recognition of the foreign-accented speech in noise than those exposed to audio-only stimuli. This replicates previous findings that audiovisual speech cues can improve recognition of accented speech in noise (Janse and Adank, 2012; Yi et al., 2013). However, we found no significant difference in the amount of perceptual adaptation between the audiovisual and audio-only groups at any stage of the experiment. If audiovisual cues were beneficial to perceptual adaptation of accented speech (in comparison to audio-only cues), we expected to observe a statistically significant difference.

## Overall Discussion

In the two studies described here, we investigated differences in perceptual adaptation to accented speech with audiovisual or audio-only stimuli. Study 1 employed an oﬄine training design and a novel accent, while participants in Study 2 were exposed to a non-native accent in either modality without separate training. In both studies, we observed a benefit from audiovisual stimuli to recognition of the accented speech in noise. However, neither study demonstrated that audiovisual stimuli can improve perceptual adaptation to accented speech when compared to audio-only stimuli; furthermore, findings from Study 2 supported the null hypothesis.

### Audiovisual Cues do not Improve Perceptual Adaptation to Accented Speech

We predicted that listeners would perceptually adapt to accented speech more when exposed to audiovisual stimuli, compared to just audio-only stimuli. We hypothesized that listeners would benefit from improved overall perception of the accented speech when visual cues were present (Arnold and Hill, 2001; Janse and Adank, 2012; Yi et al., 2013; Kawase et al., 2014), and would therefore be better able to disambiguate the unfamiliar phonetic pattern of the accent, and map it to the correct lexical items more successfully.

In Study 1, there was no significant different in adaptation between any of the groups. Bayes calculations indicated that there was indeed no effect present between the audiovisual and audio-only groups, however, much of the data was inconclusive and the training may therefore have not been fully effective. We argued that this may have been due to: (1) the length or timing of the training, (2) inconsistencies between the training and testing sessions, or (3) the specific accent or speaker. Nevertheless, after addressing these concerns in the design of Study 2, there was still no clear advantage for perceptual adaptation to accented speech with audiovisual cues. In fact, Bayes analyses suggested that the data from Study 2 support the null hypothesis – that is, the presence of visual cues does not benefit adaptation to accented speech.

Our results support previous findings by Janse and Adank (2012), who observed no significant difference in adaptation between audiovisual and audio-only accented sentences in older adults. However, our results conflict with the findings of Hazan et al. (2005), who observed that audiovisual cues *can* improve perceptual adaptation to individual non-native phonemes. These conflicting results suggest that, although audiovisual cues may help listeners to perceptually learn individual speech sounds (as in Hazan et al., 2005), this benefit does not generalize to longer items of accented speech such as sentences (as used in the present study), perhaps reflecting the increased difficulty of speech-reading longer items (Grant and Seitz, 1998; Sommers et al., 2005).

Our results suggest that perceptual adaptation to accented speech is a robust ability that is not necessarily affected by the perceptual quality of the speech, as our participants adapted to the accented speech equally in conditions with or without visual cues that improved intelligibility. Indeed, Bradlow and Bent (2008) have demonstrated that the relative intelligibility of an accent (and therefore the perceived quality of the perceptual input) does not necessarily influence the amount that listeners can adapt to it. Perceptual adaptation to accented speech may therefore be primarily driven by factors internal to the listener rather than the perceptual environment, for example statistical learning (Neger et al., 2014) or cognitive abilities (Adank and Janse, 2010; Janse and Adank, 2012; Banks et al., 2015). However, it is possible that audiovisual cues benefit listeners in ways that we did not measure in the present studies, for example in terms of listening effort – that is, the presence of audiovisual cues may have reduced the effort associated with processing accented speech (Van Engen and Peelle, 2014). A more sensitive measure such as response times may have revealed a benefit from the audiovisual cues, although this was not the case for older adults (Janse and Adank, 2012).

Some limitations to the present findings should also be acknowledged. Firstly, a benefit from audiovisual cues may be present with more exposure. Indeed, a significant benefit from audiovisual cues has been observed for perceptual adaptation to noise-vocoded speech after exposure to a greater number of stimuli than in the present two studies (Pilling and Thomas, 2011). Secondly, the audio-only group in Study 2 had a lower baseline level of recognition accuracy than the audiovisual group (15% compared to 45% accuracy); this was intentional and allowed us to confirm that the presence of audiovisual speech cues from our speaker was beneficial to performance. However, it left more room for improvement in the audio-only group and potentially impacted the amount of adaptation our participants achieved, as in both groups poorer performers adapted the most (see **Figures 6A,B**). A comparison of adaptation to audiovisual and audio-only accented speech, with baseline recognition equated in both groups, may produce different results.

### Audiovisual Cues Benefit Recognition of Accented Speech in Noise

Results from both studies replicate previous findings that audiovisual cues can benefit recognition of accented speech in noise when compared to only auditory cues (Arnold and Hill, 2001; Janse and Adank, 2012; Yi et al., 2013; Kawase et al., 2014). We observed a difference in recognition accuracy of ~30% between the two groups in Study 2, and 15% between the two groups in Study 1 (during training with background noise). It is likely that visual cues from a speaker’s facial movements help the listener to identify ambiguous or unclear phonemes by constraining the possible interpretations, or perhaps helping to identify prosodic cues (Swerts and Krahmer, 2008). Nevertheless, in both studies, we only observed greater recognition accuracy for the audiovisual groups when background noise was present, suggesting that benefits may have been related to compensation for the background noise, rather than the accented speech *per se*. Particularly, in Study 1 we did not observe a significant difference in recognition accuracy between the audiovisual and audio-only training groups when the stimuli were presented in quiet. However, recognition accuracy for these training groups was almost at ceiling level and the additional perceptual input from the audiovisual cues may therefore have been redundant, as the perceived clarity of the auditory signal can influence the benefits gained from audiovisual speech cues (Ross et al., 2007).

Listeners can perceptually adapt to accented speech very rapidly, even after exposure to a few sentences (cf. Clarke and Garrett, 2004), and this poses a practical limitation to studies of perceptual adaptation to, or recognition of, accented speech. As in the present studies, the most commonly used method to avoid ceiling effects is to add background noise, and this is the context in which an audiovisual benefit to accented sentences has previously been observed (Janse and Adank, 2012; Yi et al., 2013). However, two studies have also demonstrated this effect with audiovisual stimuli presented in quiet. Kawase et al. (2014) investigated adaptation to audiovisual accented phonemes in quiet; however, removing any lexical or semantic information increases the task difficulty, but perhaps does not reflect an ecologically valid context. Arnold and Hill (2001) used longer speech passages and a semantic comprehension task to assess the contribution of audiovisual cues; but, the task may have reflected semantic memory processes rather than speech recognition *per se*, and the result has not since been replicated. The extent to which audiovisual cues can benefit recognition of accented speech in optimal, quiet listening conditions remains, therefore, to be confirmed.

Finally, we observed different amounts of audiovisual benefit between the two studies. This may be explained by differences in the speaker and accent used. Kawase et al. (2014) observed that audiovisual speech affects the perception of non-native phonemes to varying degrees; it is therefore likely that different accents result in varying benefits from visual speech cues. Furthermore, visemes (the visual equivalent of phonemes) from different speakers can vary in intelligibility (for example, Kricos and Lesner, 1982, 1985), possibly resulting in different benefits from our two speakers. Our results therefore add to existing evidence that being face-to-face with a speaker does not always benefit the listener to the same extent.

## Conclusion

The present studies demonstrate that audiovisual speech cues do not benefit perceptual adaptation to accented speech – that is, observing audiovisual cues from a speaker’s face does not lead to greater improvements in recognition of accented speech over time, when compared to listening to auditory speech alone. Audiovisual cues may still provide benefits to recognition of accented speech in noisy listening conditions, as we found a benefit to recognition of both types of accented speech in noise in comparison to audio-only speech. However, our results also demonstrate that the benefits obtained from audiovisual speech cues vary greatly, and the extent to which they benefit recognition of accented speech, as opposed to background noise, still needs to be clarified.

## Conflict of Interest Statement

The authors declare that the research was conducted in the absence of any commercial or financial relationships that could be construed as a potential conflict of interest.

## Footnotes

1 In normally distributed data, z-scores would not be expected to be greater than 3.29.
